# Micro-nano hybrid structures with manipulated wettability using a two-step silicon etching on a large area

**DOI:** 10.1186/1556-276X-6-333

**Published:** 2011-04-14

**Authors:** Beom Seok Kim, Sangwoo Shin, Seung Jae Shin, Kyung Min Kim, Hyung Hee Cho

**Affiliations:** 1 Department of Mechanical Engineering, Yonsei University, 262, Seongsanno, Seodaemun-gu, Seoul 120-749, Korea

## Abstract

Nanoscale surface manipulation technique to control the surface roughness and the wettability is a challenging field for performance enhancement in boiling heat transfer. In this study, micro-nano hybrid structures (MNHS) with hierarchical geometries that lead to maximizing of surface area, roughness, and wettability are developed for the boiling applications. MNHS structures consist of micropillars or microcavities along with nanowires having the length to diameter ratio of about 100:1. MNHS is fabricated by a two-step silicon etching process, which are dry etching for micropattern and electroless silicon wet etching for nanowire synthesis. The fabrication process is readily capable of producing MNHS covering a wafer-scale area. By controlling the removal of polymeric passivation layers deposited during silicon dry etching (Bosch process), we can control the geometries for the hierarchical structure with or without the thin hydrophobic barriers that affect surface wettability. MNHS without sidewalls exhibit superhydrophilic behavior with a contact angle under 10°, whereas those with sidewalls preserved by the passivation layer display more hydrophobic characteristics with a contact angle near 60°.

## Introduction

In general, boiling heat transfer is considered to be very effective mechanism for cooling the high heat-generating devices due to the large latent heat by phase transition accompanied by fast transport of gas-phased bubbles. In many industrial fields related to energy conversion, e.g., nuclear power plants, heat pump, and electronics, improving the performance of boiling heat transfer based on surface treatment and modification is a key issue [1-3]. Both microstructures and nanostructures are often used to enhance the performance of boiling heat transfer by controlling and modifying structure geometries.

There have been numerous studies on boiling heat transfer improvements obtained by microscale structure fabrication using artificial structures, such as patterned circular/rectangular holes/pillars, and conical/cylindrical cavities [4,5]. With the development of feasible nanoscale fabrication technique including nanostructure patterning by conventional photolithography [6] or maskless method [7] that can easily manipulate the surface wettability [8,9], nanoscale surface treatments could also be applied to boiling heat transfer enhancement. 3D macro-porous metallic surface layer with nanoscale porous structures-enhanced heat transfer coefficient, especially at low heat flux of 1 W · cm^-2^, over 17 times compared to the plain surface [10]. Top-down etched silicon nanowires (SiNWs) and electrodeposited copper nanowires improved the boiling performance by up to 100% compared to a plain silicon surface, by increasing surface wettability where the nanowires exhibited superhydrophilic behavior [11]. With tilted copper nanorods, synthesized by an electron-beam evaporator, pool boiling heat transfer characteristics were also enhanced by the increased wettability and the nucleation sites resulting from the intrinsic nature of the dense nanowires [12]. Additionally, some studies report the increase in boiling behavior using carbon nanotube-coated surfaces [13]. It may be inferred from these references that nanoscale structures greatly increase the surface area and wettability and lead to the enhancement of boiling behavior by supplying adequate liquid to the boiling surface and extending the burn-out limit of the surfaces.

In light of previous efforts to enhance boiling performance by increasing the nucleation sites and the surface wettability, micro-nano hybrid structures (MNHS) [14,15] may offer extraordinary boiling heat transfer performance. Specifically, hierarchical MNHS can significantly increase the boiling surface area, the surface roughness and the surface wettability, compared to single-scale structures. However, there has been relatively little research on the fabrication and the application of hierarchical MNHS to enhance the performance of boiling heat transfer further.

For the fabrication of MNHS, nanowire-adorned microstructures by selective electrochemical growth of nanowires [14], using a porous anodic alumina template [16] and the dual-scale hierarchical structures with SU-8 photoresist (PR) using capillary force lithography [17] were reported. However, for boiling applications there are some specialized requirements which should be met in prior. First, the boiling surface must have good thermal properties, including high thermal conductivity and durability under high heat flux conditions. Second, the surface area and roughness should be increased further by modifying surface geometries, for example, combining microscale patterns with nanoscale structures, to expel the heat from the surface sufficiently and to act as a bubble nucleation site. Third, the fabrication technique must be simple, and must enable one to simultaneously synthesize nanoscale structures and microstructures over a large area. Fourth, a hydrophilic surface, which can readily attract and supply the cooling agent to the boiling surface, is desirable to prevent a film formation on heated surface for boiling applications. In view of these requirements, previous MNHS fabrication techniques, which were complicated by the use of templates and additional electrode layers, or were intended for low thermal conductivity and hydrophobicity based on polymer materials, may be inappropriate for boiling applications.

In this study, we focus on the hierarchical structure formations by fabricating hierarchical MNHS that meet the boiling heat transfer requirements mentioned above. In particular, we propose a simple fabrication process using two-step silicon etching: silicon deep trench reactive ion etching (DRIE) for microstructure fabrication and electroless silicon etching for nanowire formation. These processes are feasible and robust. In particular, the electroless silicon etching process enables uniform nanowires to be readily fabricated over a large area at room temperature, without any catalysts or templates [18-20]. By this simple technique, we fabricated wafer-scale hierarchical MNHS made up of micropillars/cavities covered with uniformly grown nanowires. By controlling the removal of natively coated polymeric passivation layers during DRIE (or Bosch process) [21], we obtained various combined structures by two-step silicon etching process. Especially, by removing the polymeric passivation layers which induce the conglomeration of nanowires at the boundary of micropatterns and thus make hydrophobic surface by prohibiting the cooling agent from spreading or wicking, hierarchical MNHS can readily serve to pump the water cooling agent to the surface due to the superhydrophilic characteristics. We validated that the surface wettability of those surfaces with hierarchical MNHS by measuring the surface contact angle for deionized water. Based on our results, we believe that the surfaces with hierarchical MNHS may be candidates for boiling heat transfer applications.

## Experimental section

### Sample preparation: top-down SiNWs

**Figure 1:**
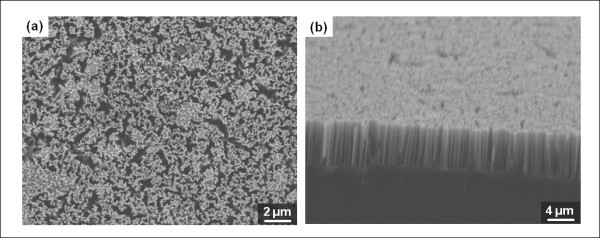
**SEM images of synthesized SiNWs by the electroless etching method**: (a) a top view of nanowire surface; (b) a cross-sectional view of the nanowires.

Using electroless metal deposition and anisotropic silicon etching, SiNWs with very high aspect ratios can readily be synthesized on a wafer-scale area of a silicon substrate. We refer to previous studies for detailed electrolyte recipes and fabrication processes [11,19,20]. Prior to the formation of micropatterns and nanowires, the silicon wafer was thoroughly cleaned. Here, we used an n-type silicon wafer (phosphorous-doped) with a (100) orientation, a resistivity between 1 and 10 Ω cm, and a thickness of 500 μm. To begin with, a 4-inch silicon wafer was cleaned for 40 min in an H_2_O_2 _and H_2_SO_4 _solution with a volume ratio of 1:3, to remove organic materials. This was followed by an additional cleaning process with acetone and methanol for 5 min each in turn, using a sonicator. For fabricating SiNWs by electroless etching, the wafer was immersed in an aqueous solution of 0.02 M AgNO_3 _and 5 M HF for 70 min at room temperature. When diluted AgNO_3 _and HF solution are used for electroless silicon etching, Ag^+ ^ions are attracted to the silicon surface by galvanic displacement. At the interface between the Ag^+ ^and the silicon, oxidization of the silicon takes place, and, subsequently, the oxidized layer is etched by the hydrofluoric acid. Throughout the electroless silicon wet etching process, SiNWs were uniformly formed on the whole wafer surface, and silver dendrites covered the entire substrate [15]. After silicon etching, the wafer was immersed in HNO_3 _solution (70%) for 80 min to remove the silver dendrites and reveal the nanowire arrays. Finally, the sample was thoroughly rinsed with deionized water and dried under ambient conditions. Figure 1 shows field emission scanning electron microscope (FE-SEM) images of nanowires aligned vertically on a silicon substrate. In our fabrication process, the lengths and diameters of the nanowires were about 10 μm and 100 nm, respectively.

### MNHS fabrication using two-step silicon etching process

**Figure 2:**
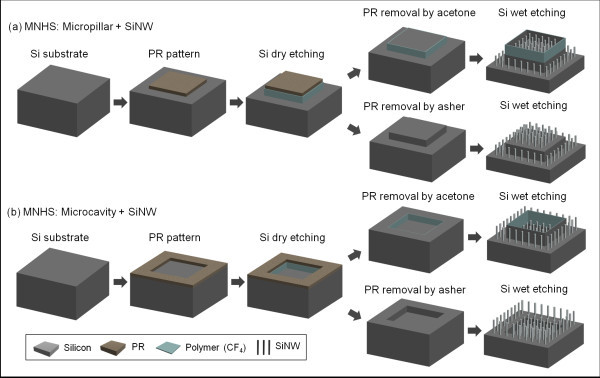
**Schematics of the fabrication processes for MNHS**: (a) MNHS with micropillar and nanowires using acetone-based PR stripping and asher-mediated process; (b) MNHS with microcavities and nanowires using acetone-based PR stripping and asher-mediated process.

To fabricate the MNHS with micropillars or microcavities, we used a two-step silicon etching process, consisting of dry etching (DRIE) and wet etching (electroless etching). The processes for MNHS with micropillars/microcavities with SiNWs are outlined schematically in Figure 2a,b, respectively. First, the silicon was dry etched to fabricate the microstructures. In the general silicon DRIE process (Bosch process) that is widely used as a dry etching method for fabricating deep silicon trenches, a polymeric C_4_F_8 _passivation layer is precoated to prevent over-etching of the sidewalls of the silicon patterns. This can be left in place or removed from MNHS by choosing the appropriate PR stripping method. MNHS with thin sidewall structures are fabricated by removing the PR layer with acetone, which leaves the polymeric passivation layer intact. On the other hand, to fabricate MNHS with no thin sidewall structures, the PR and polymeric passivation layer deposited on the sidewalls are removed by the asher process.

### Surface characterization

For the characterizations in this study, FE-SEM images were taken by JEOL-JSM-6700F scanning electron microscope. In addition, determining the polymeric residuals on the sidewalls of MNHS were also performed by energy dispersive spectrometer (EDS) equipped with the same SEM.

### Contact angle measurement

All measurements of surface contact angle were conducted using KSV CAM-200 (KSV Ins.). The value of contact angle on each fabricated substrate was automatically calculated based on the calibrating program, KSV Contact Angle Measurement System. We used DI water droplet having volume of 2 μl and captured droplet images with frame interval of 2 ms using CCD camera with resolution of 512 × 480 pixels. The presented values of contact angle in this article are averaged value obtained by measurements more than three times on the same substrate but not on the same local spot.

## Results and discussion

### Micro-nano hybrid structures: micropillars and nanowires

**Figure 3:**
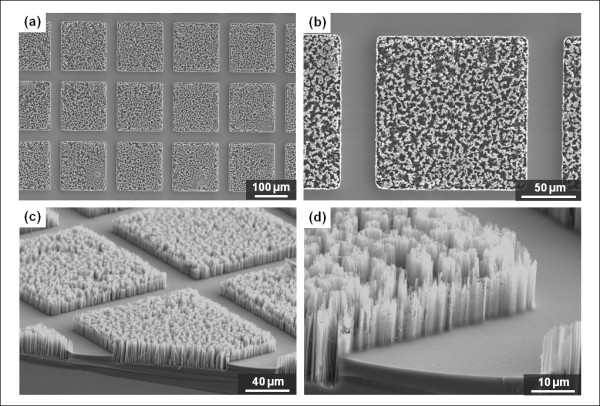
**MNHS with micropillars fabricated with acetone-based PR stripping**: (a, b) top-view images; (c, d) tilted-view images. The width of a square micropillar and the gap between pillars are 100 and 20 μm, respectively.

By sequentially using the DRIE technique for micropatterns and electroless etching of silicon for nanowires, we can hierarchically design MNHS with micropillars and nanowires. Figure 3 shows SEM images of the fabricated structures. Here, the width of a square micropillar and the gap between pillars are 100 and 20 μm, respectively. As can be seen from these figures, the nanowires were well synthesized over the entire area, including the tops of the pillars and the trench regions.

**Figure 4:**
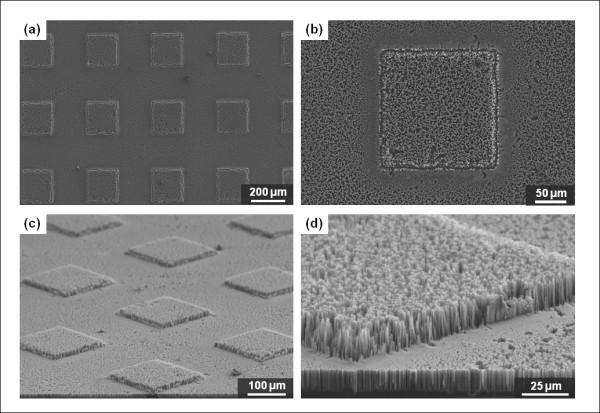
**MNHS with micropillars using asher-mediated process**. (a, b) top-view images; (c, d) tilted-view images. The width of a square micropillar and the inter-pillar distance (gap distance between pillars) are both 200 μm.

Because the silver ions attach themselves to the surface of the silicon, and a thin C_4_F_8 _polymer layer is deposited on the sidewalls of micropatterns during the silicon dry etching process for passivation [21], we can manipulate the nanowire-formation characteristics at the boundaries of the micropillars. Specifically, the polymer layer deposited on the sidewalls of the micropatterns during DRIE may also act as a mask for the SiNWs etching. Following DRIE, PR stripping is accomplished using acetone or the microwave plasma asher process. Liquid acetone can only remove the PR layer, while the asher process (which uses oxygen plasma) can remove both the PR and the sidewall polymer. Figure 3 shows the thin sidewalls that were formed by conglomeration of nanowires and undamaged silicon surrounding the boundaries of micropillars. Using the asher process after the micropattern fabrication, it is possible to retain a more porous structure without thin sidewall silicon barrier. In Figure 4, one can clearly see that sharp, thin walls are not formed at the boundaries of the micropillars, whereas they are wholly formed from Figure 3. Using an EDS for MNHS fabricated by the acetone process, we confirmed that small quantities of C and F remained on the sidewalls of the micropillars. On a sidewall of MNHS fabricated by acetone-treated process, C and F were detected by 10.83 and 0.42 wt%, respectively. On the other hand, there were not any elemental compositions of polymeric passivation layer on the sidewalls of MNHS by asher-treated process. This explains the existence of the polymeric C_4_F_8 _passivation layer that covers the sidewalls and acts as a protective layer against the etching solution.

### Micro-nano hybrid structures: microcavities and nanowires

**Figure 5:**
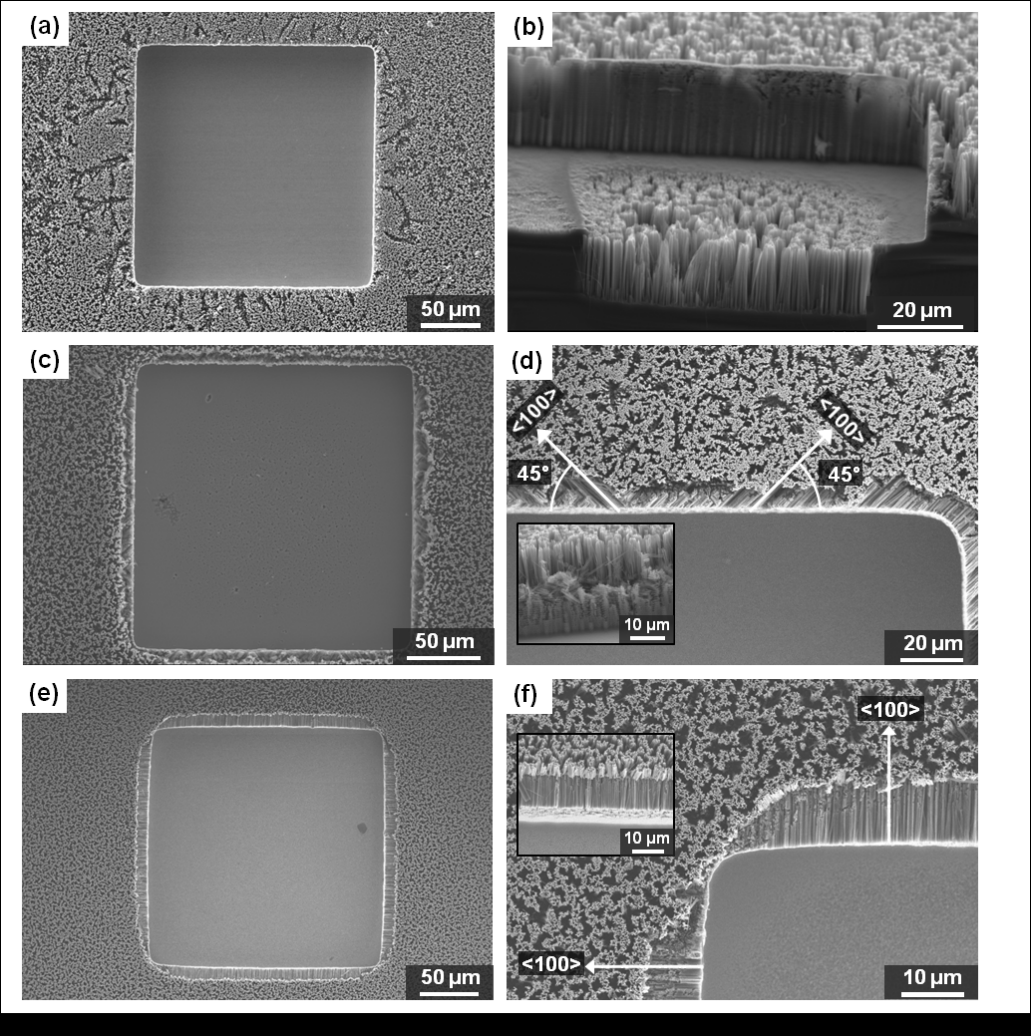
**MNHS with microcavities having a hole width of 200 μm and distance between cavities of 200 μm**: (a, b) acetone-mediated MNHS. The thin sidewall structure is clearly shown; (c, d) asher-mediated MNHS without any substrate rotation. The inset in (d) is a close-up image of the side region; (e, f) with 45° rotation of the substrate. The inset in (f) is a top view of laterally grown nanowires on the sides of the microcavities.

Figure 5 shows SEM images of microscale square cavities with nanowires. For the microscale cavities, the width and the depth of a square, and the distance between cavities is 200, 30, and 200 μm, respectively. Using the acetone wet-cleaning process instead of the asher process, it is also possible to make a thin sharp sidewall barrier that surrounds the microcavity structures. Figure 5a,b shows a fabricated structure with thin sidewalls. However, it should be noted that the specimens shown in Figure 5c,d,e,f was fabricated using the asher process to remove the polymeric passivation layer. By contrast, unlike the previous structures (Figure 5,b), when we fabricated MNHS using the asher process instead of the acetone process after the silicon dry etching, no thin, sharp sidewalls are observed at the boundaries of the microcavities. The boundaries are also etched out by 1-h silicon wet etching in diluted HF and AgNO_3 _solution for nanowire synthesis. From the top view shown in Figure 5c,d,e,f, we can infer that the bulk portion of the silicon near the boundary area of the square microcavities could have been etched away, because of the orthogonal silicon crystallographic orientation. In the inset of Figure 5d, which shows a cross-sectional view of the boundary area, we observe that nanowires with specific angles of ± 45° to the flat plane are formed on the sidewall of the microcavity. According to previous studies [22-24], in electroless silicon etching, SiNWs are synthesized with a certain orientation, determined by the crystallographic orientation of the wafer. They reported that SiNWs are synthesized primarily in the normal direction with a (100) wafer crystallographic orientation. In our fabrication process, we used (100) Si wafers and the white arrow lines in Figure 5c,d,e,f indicate the in-plane 〈100〉 direction. Because the silicon is etched parallel to the 〈100〉 orientation, silicon etching from the sidewall and top surface must eventually intersect during the etching process, and thereby the silicon near the boundary will be etched away at the same time. Figure 5e,f shows images of the structure fabricated on the substrate with 45° rotation. As expected, the nanowires on the sidewall surface are normal to the boundaries that are parallel to the (100) substrate crystallographic orientations.

However, nanowires are locally formed at the bottoms of the microholes. As the cross-sectional view of Figure 5b shows, the nanowires were fabricated in the center region of the bottom surface, but do not appear on the surface near the sidewalls. Additional fabrications were carried out, but very little uniformity, symmetry, or repeatability was noted in the fabricated nanowires on the bottom surfaces. During the electroless etching process, we have often observed that when the etching solution is poured over the silicon substrate, air bubbles are initially formed on the square cavity patterns. As the etching continues for over an hour, these bubbles may decay. It may be difficult for the etching solution to make contact with the bottom surface of the microcavity, and for silver ions to adhere to that surface. Moreover, even if silver ions initially manage to attach themselves to the surface, as the etching progresses, it becomes increasingly difficult to replenish the etching solution to etch the oxidized silicon layer (SiO_2_) under the attached silver particles on the silicon through the silver dendrites that cling to the surface and spread over the whole silicon substrate. During the electroless silicon etching process, we did not use any artificial stirrer or sonicator to mix and supply the solution near the etching surface or to destroy the initial air bubbles inside the microcavities.

### Surface wettability and potential for boiling applications

**Figure 6:**
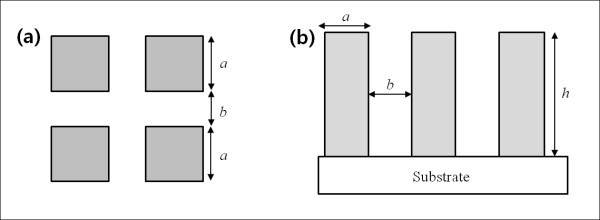
**Schematics of pillar structures on a substrate**: (a) top-view; (b) side-view.

In this study, for the bare Si substrate which shows a hydrophilic behavior with contact angle less than 90° (*θ*_avg _= 70.1° like Figure 6a in the manuscript), surface wettability could be explained by Wenzel's relationship. Considering surface roughness by the geometrical characteristics, contact angle on the surface could be described by the following equation from reference of:(1)

where *θ *is the apparent contact angle actually measured on the surface, θ* is the equilibrium contact angle on an ideal plain surface, and *r *is the roughness factor, defined as the ratio of the actual surface area to the projected one. If we imagine rectangular-pillar structures on a substrate like Figure 6 with pillar width, *a*, spacing between pillars, *b*, and height, *h*, we can express roughness factor as follows:(2)

In the light of microscale structures (not nanoscale ones), for example, *r *should be increased by increasing *h *then the surface would be more hydrophilic by decreasing surface contact angle. In addition, when the spacing between the pillars (*b*) decreases on structures with fixed width (*a*) and height (*h*), contact angle also decreases due to the increased roughness factor.

**Figure 7:**
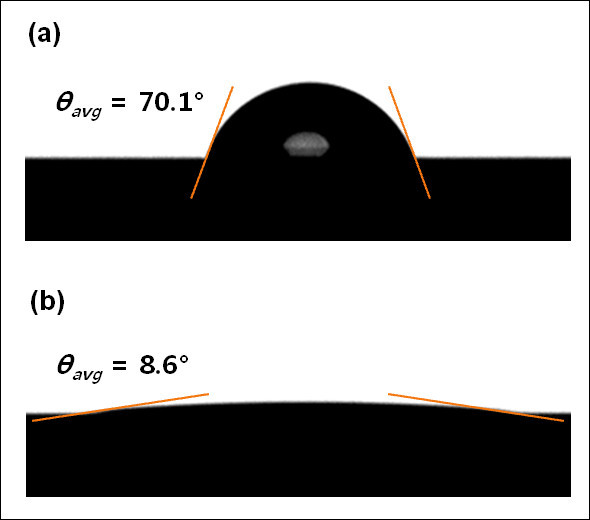
**Contact angle (*θ*) measurements for the fabricated silicon surfaces**: (a) n-type bare silicon following the entire cleaning process; (b) silicon covered with nanowires.

However, if we consider the silicon nanowires synthesized on the overall surfaces in this study, they would induce extremely high surface roughness due to their nanoscale dimensions. Figure 7 displays the contact angle measurements for a bare silicon surface along with the surface having nanowires. Our measurements suggest that a nanowire-coated surface becomes highly hydrophilic at contact angles less than 10°. Once the liquid droplet falls on the surface, the contact angle comes to superhydrophilic near 0 degree by wicking. A silicon surface with dense nanowires has high water-wettability, which results in a very low contact angle at the solid and liquid interface. This is in close agreement with previous experimental results [11] and theoretical predictions [25] for nanowire-coated surfaces. In view of the roughness effect on the contact angle *θ*, for a hydrophilic n-type silicon surface (*θ*_silicon _= 70°) [26], nanowires having very high aspect ratio induce highly hydrophilic behavior by increasing the surface roughness [25,27]. The wicking condition in porous structure can be explained by the criteria for the surface energy based on the geometry [25]:(3)

where *θ*_c _and *φ*_S _indicate the criteria contact angle to induce an intermediate condition between the water spreading and the imbibition through the surface (*θ *= 0 and *θ *<*π*/2, respectively), and the solid fraction remaining dry, respectively. When we assume that the nanowires with 10 μm length have the width of an edge and the distance between nanowires are both 100 nm, the rough surface with the nanowires readily satisfies the criteria for wicking and thus leads to superhydrophilic characteristics.

These results reveal that MNHS totally covered by nanowires as well as the plain surface with nanowires could be superhydrophilic and effective to increase the liquid wicking effect. On the other hand, for hybrid structures fabricated by the acetone-based PR-removal process, the thin solid sidewalls may prohibit the liquid from spreading throughout the nanowires. The polymer residuals remaining on the sidewalls of MNHS tend to propel liquid water because of their intrinsic hydrophobicity. For reasons such as these, the contact angles of MNHS with sidewall structures are strikingly larger than those without sidewalls. Table 1 shows comparative results for MNHS surfaces with or without thin and sharp sidewall structures coated by polymeric passivation layer. Here, the contact angle measurements were conducted for the microstructures with size of 100 μm × 100 μm. However, though MNHS have the thin sidewall structures, MNHS after the additional asher process to remove the C_4_F_8 _layer from the walls are shown to be superhydrophilic. This clearly explains the effect of passivation layer for the unusual wettability characteristics.

**Table 1:** Contact angle (*θ*) measurements for the MNH**S**.

Figure^a^	Micro-pattern	PR removal	Contact angle
	Pillar	Asher	
	Pillar	Acetone	
	Cavity	Asher	
	Cavity	Acetone	

^a^SEM images displayed do not match practical sizes, but present geometrical structures.

**Figure 8:**
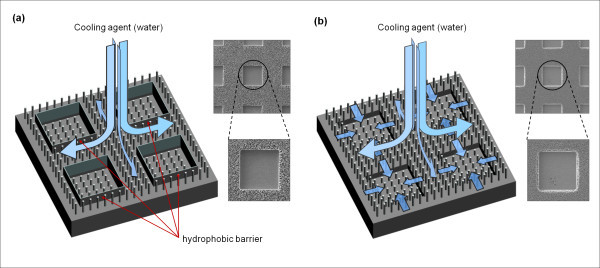
**Schematics for behavior of water cooling agent on the surfaces with MNHS**: **(a) **MNHS with microcavities and nanowires fabricated by acetone-mediated process; **(b) **MNHS with microcavities and nanowires fabricated by asher-mediated process to remove the hydrophobic barrier structure.

The heat transfer area is significantly increased by the additional microscale pattern structures in hierarchical MNHS than the surface with just nanoscale structures. Moreover, microscale patterns designed in accordance with vapor size (tens or hundreds of microns in a boiling process) and porous vacancies between nanowires can also play a role as artificial bubble nucleation sites in boiling application [12,28]. In addition, by controlling the sidewall structure formation during the fabrication of MNHS and then removing the polymeric passivation layer on it, it is possible to control the surface wettability, and thereby the boiling performance. Using the asher process to remove the passivation layer deposited during DRIE process, nanowires are densely synthesized over an entire area, resulting in very high surface wettability. High surface wettability efficiently pumps and supplies water to the boiling surface, which extends the surface burn-out limit (critical heat flux, CHF limit) [11,12]. On the other hand, MNHS fabricated with polymer-passivated sidewalls may decrease the boiling performance by their hydrophobic behavior. Figure 8 explains the behavior of water cooling agent on those surfaces with MNHS. Surface wettability affects the bubble generating and detaching schemes from heat-emitting surfaces [29]. The hydrophobic sidewalls fabricated by the acetone process prohibit water from coming through the porous structures towards the artificial micropatterns as nucleation sites. Then the cooling efficiency would be decreased by merged vapors that would not be detached from the surface by insufficient refreshing water and suppress heat emission, and this finally leads to the burn-out of the local areas. However, hierarchical MNHS having highly hydrophilic characteristics can supply and refresh water to the initiative bubble generating regions more collaboratively than one having hydrophobic barrier structures. Thus, the burn-out of the surface and film boiling can be retarded according to the increasing of a CHF limit [30]. Based on these wicking mechanisms as shown in Figure 8, boiling performance could be improved by MNHS that are accompanied by the geometrical combination of micro- and nanoscale structures and the superhydrophilic surface wettability.

## Conclusions

We fabricated hierarchical MNHS using a two-step silicon etching process, consisting of dry etching (DRIE or Bosch process) for micropattern formation and electroless silicon wet etching for synthesizing nanowires. MNHS that lead to geometrically rough and superhydrophilic advantages for boiling application form hierarchical structures having micropillars or microcavities with high-aspect-ratio nanowires. The fabrication process is simple and cost effective, and can readily produce MNHS over the area of an entire wafer. By controlling polymer-removal technique, we can create an artificial surface with microscale nucleation sites favorable for bubble generation in boiling heat transfer. Specifically, MNHS will have a conspicuously large boiling surface area and superhydrophilic characteristics. It will improve the boiling performance over a broad heat flux range by introducing hydrophilic regions, removing hydrophobic sidewall structures that are usually formed in silicon dry etching process. MNHS having superhydrophilic characteristics can supply and refresh water to the surface more collaboratively than one having hydrophobic barrier structures. Thus, the burn-out of the surface can be retarded according to the increasing of a CHF limit and preventing film boiling. In view of these characteristics, boiling performance could be improved by MNHS that are accompanied by the geometrical combination of micro- and nanoscale structures and the superhydrophilic surface wettability. A design study for optimal MNHS, as well as an experimental evaluation of the performance of boiling heat transfer should be topics for future research, and are currently under investigation.

## Abbreviations

CHF: critical heat flux; DRIE: deep trench reactive ion etching; EDS: energy dispersive spectrometer; FE-SEM: field emission scanning electron microscope; HMDS: hexamethyldisiloxane; MNHS: micro-nano hybrid structures; PR: photoresist; SiNWs: silicon nanowires.

## Competing interests

The authors declare that they have no competing interests.

## Authors' contributions

BSK carried out conception, fabrication of MNHS, surface characterizations for geometries and surface wettability, and drafted the manuscript as the first author. SS also carried out conception for this study and surface characterizations using FE-SEM and participated in draft writing. SJS conducted practical MNHS fabrication, surface wettability characterizations, and graphical information creation and participated in draft writing. KMK carried out conception and evaluated the application of this study for boiling heat transfer enhancement. HHC conceived of this study as a corresponding author and performed conception of MNHS, evaluated possibility for heat transfer applications and corrected the manuscript. All authors participated in the draft writing and approved the manuscript before the submission in *Nanoscale Research Letters*.
